# Supplementary figures and images for: Establishment and characterization of a new immortalized human adenomyosis epithelial-like cell line, tAEC21
Source: Biol Reprod. 2025 Nov 20;114(4):1258–76. doi: 10.1093/biolre/ioaf255 (PMC13079455; doi:10.1093/biolre/ioaf255)

Supplemental Figure S1, Klymenko *et al.*

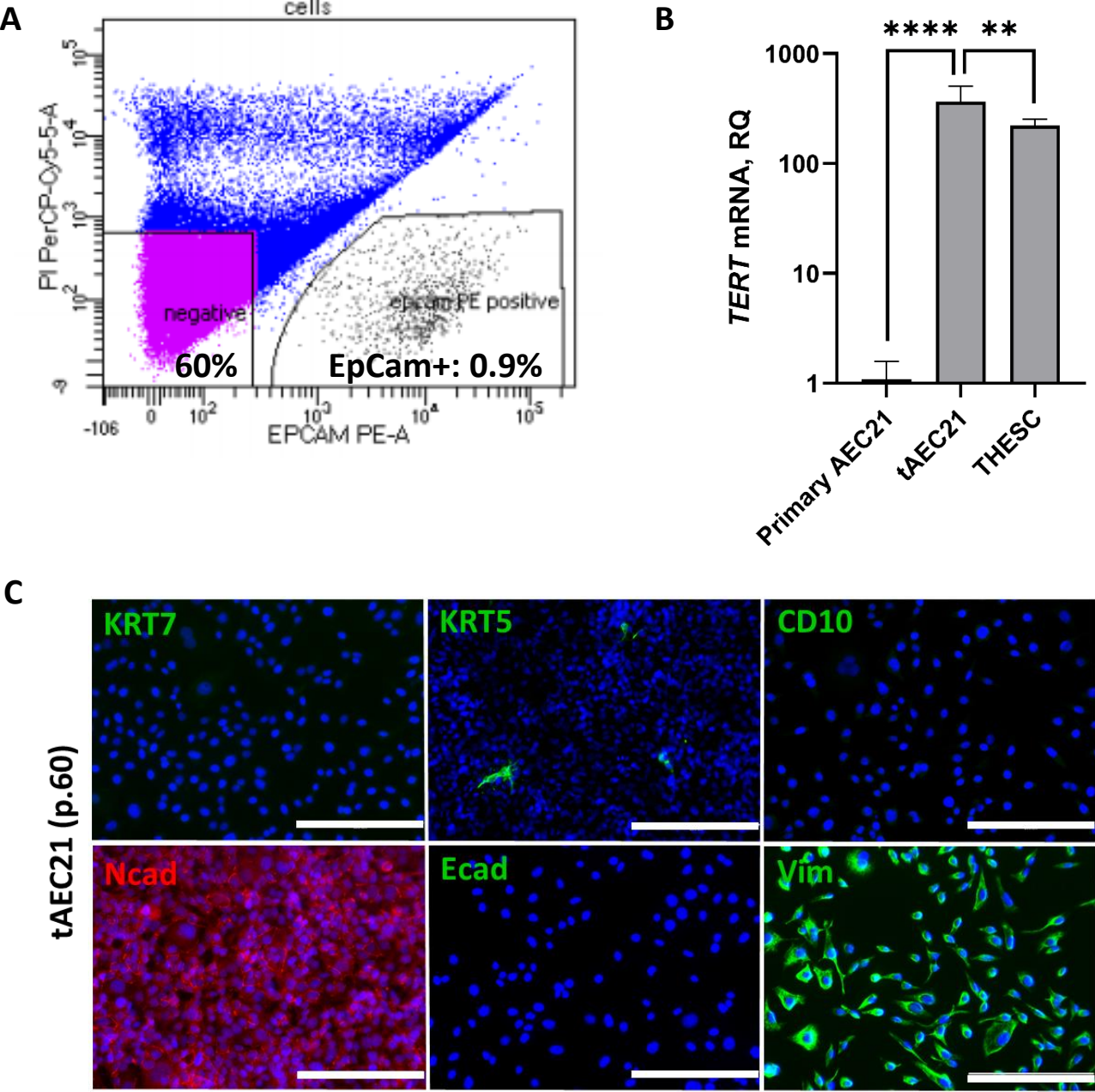

Supplement: Suppl_Figure_S1_ioaf255 [file suppl_figure_s1_ioaf255.pdf]

A

Passage 6

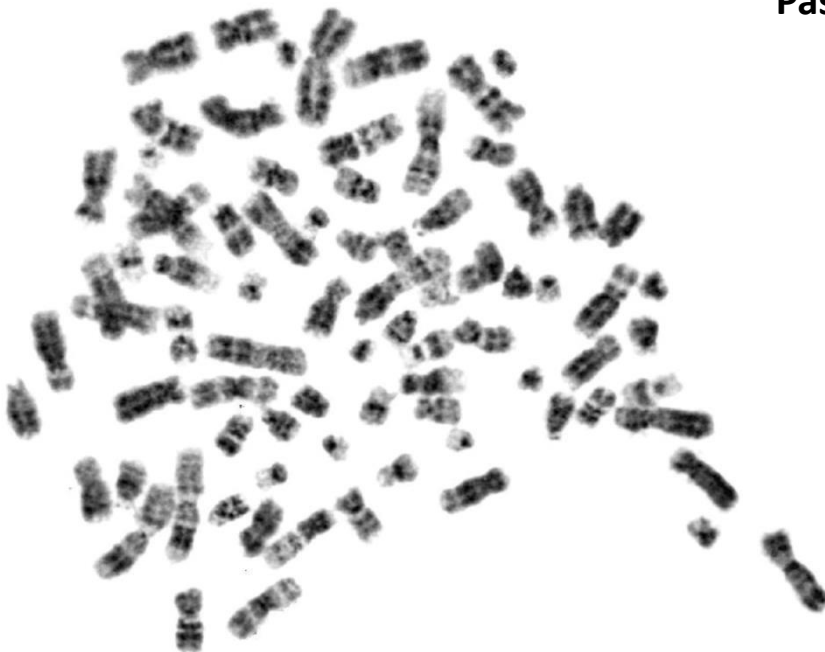

B

Passage 53

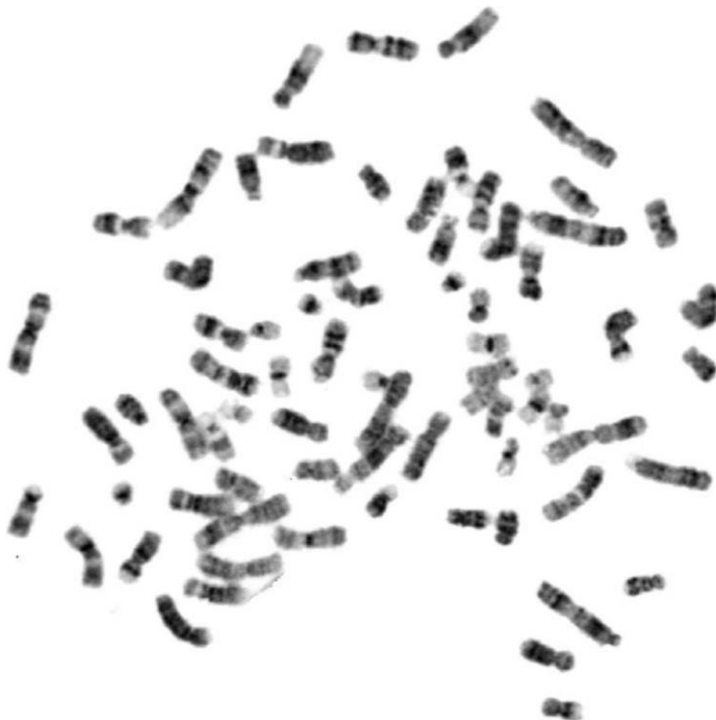

Supplement: Suppl_Figure_S2_ioaf255 [file suppl_figure_s2_ioaf255.pdf]

Supplemental Figure S3, Klymenko *et al.*

A

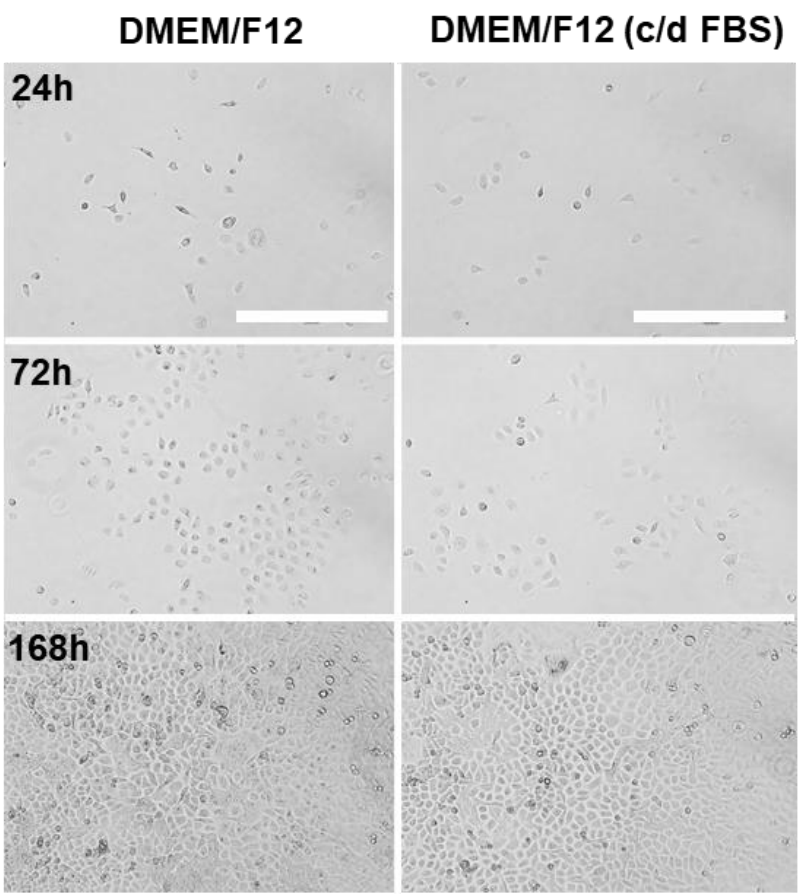

B

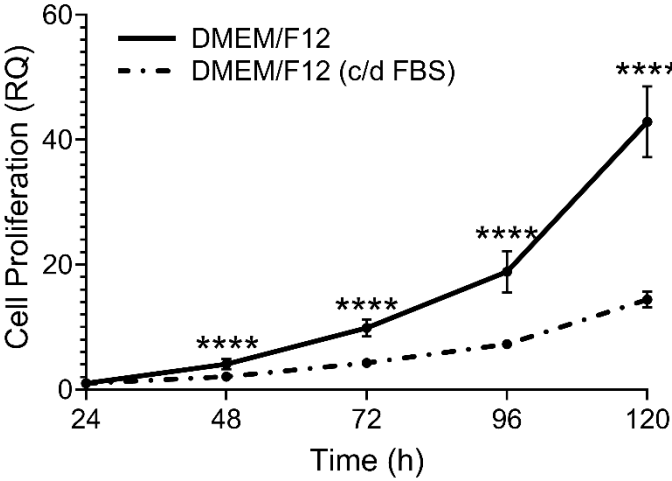

C

| Media              | Doubling Time (h) |
|--------------------|-------------------|
| DMEM/F12           | 21.18             |
| DMEM/F12 (c/d FBS) | 25.89             |

D

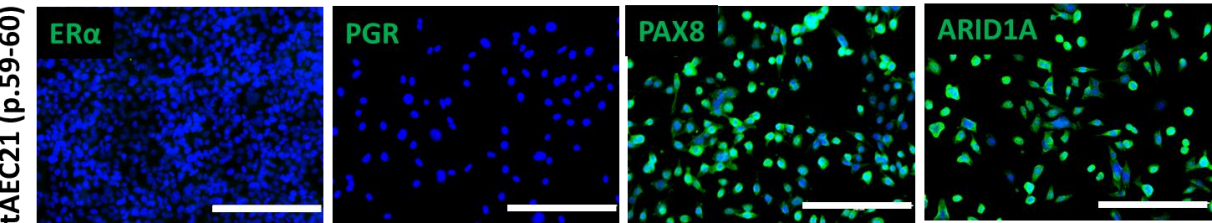

Supplement: Suppl_Figure_S3_ioaf255 [file suppl_figure_s3_ioaf255.pdf]

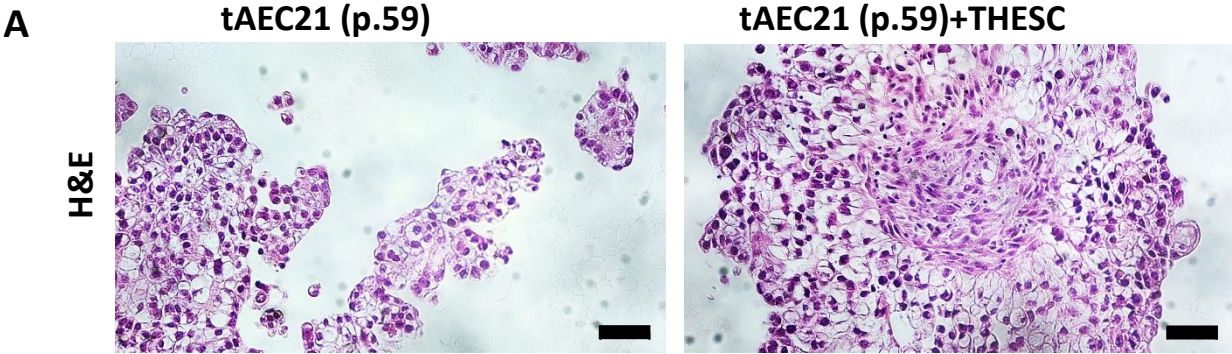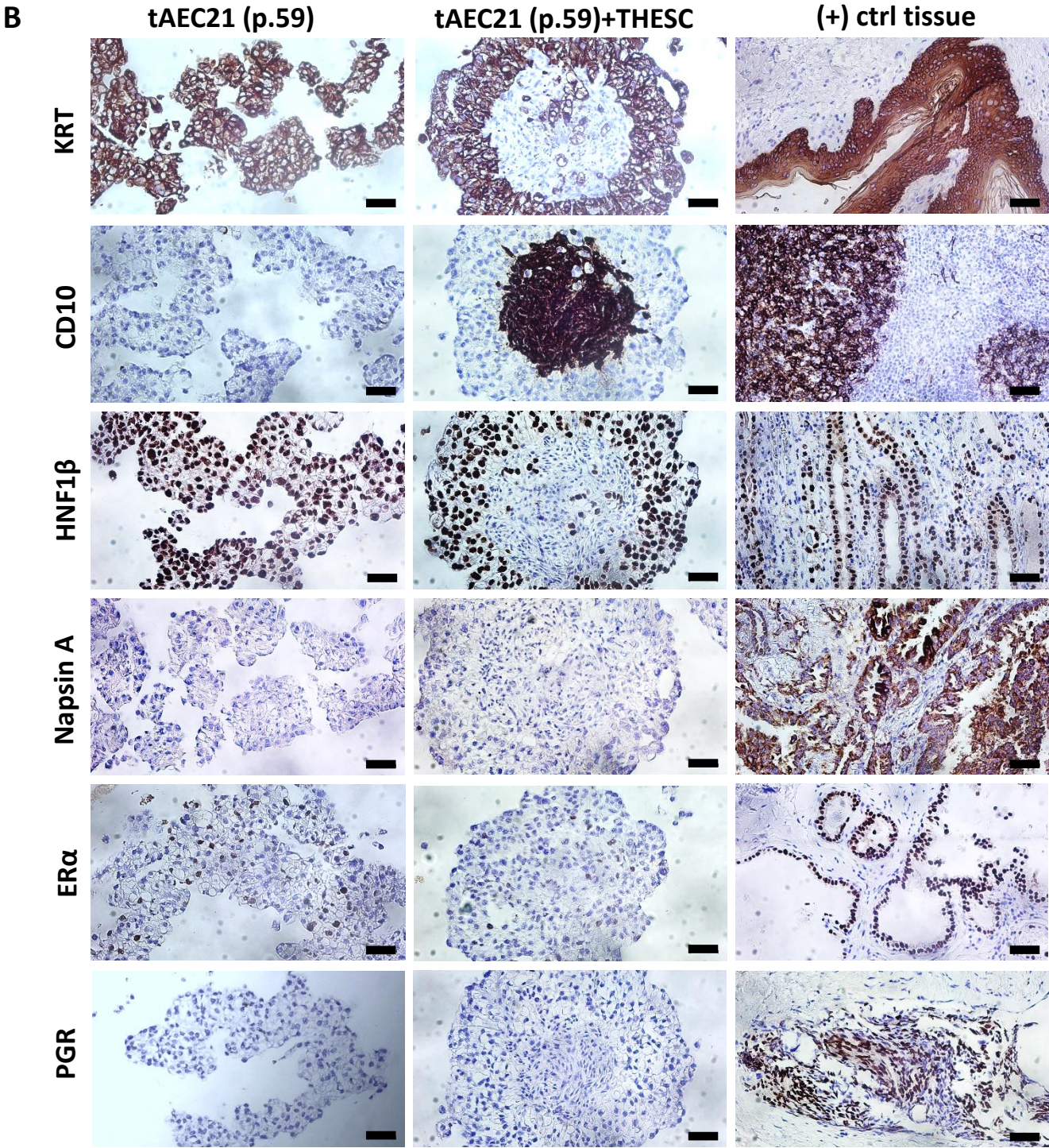

Supplement: Suppl_Figure_S4_ioaf255 [file suppl_figure_s4_ioaf255.pdf]
