# Supplementary material for: Establishment and characterization of a new immortalized human adenomyosis epithelial-like cell line, tAEC21
Source: Biol Reprod. 2025 Nov 20;114(4):1258–76. doi: 10.1093/biolre/ioaf255 (PMC13079455; doi:10.1093/biolre/ioaf255)
Supplement: Suppl_Table_S2_STR_marker_profile_ioaf255 [file suppl_table_s2_str_marker_profile_ioaf255.docx]

**Table 1.** IDEXX CellCheck^TM^16 established a unique 16 short-tandem repeat (STR) marker profile for tAEC21 cell line

| **Marker Name** | **tAEC21 Genetic Profile Matches** |
| --- | --- |
| *AMEL* | X |
| *CSF1PO* | 11 |
| *D13S317* | 8, 10 |
| *D16S539* | 11, 12 |
| *D18S51* | 16, 18 |
| *D21S11* | 31, 31.2 |
| *D3S1358* | 14 |
| *D5S818* | 11, 12 |
| *D7S820* | 11, 12, 13, 14 |
| *D8S1179* | 14, 15 |
| *FGA* | 23, 24, 25, 26 |
| *Penta_D* | 12, 13 |
| *Penta_E* | 5, 13 |
| *TH01* | 9, 9.3 |
| *TPOX* | 8, 11 |
| *vWA* | 18, 19 |
